# Trends in Incidence of Metastatic Prostate Cancer in the US

**DOI:** 10.1001/jamanetworkopen.2022.2246

**Published:** 2022-03-14

**Authors:** Mihir M. Desai, Giovanni E. Cacciamani, Karanvir Gill, Juanjuan Zhang, Lihua Liu, Andre Abreu, Inderbir S. Gill

**Affiliations:** 1 USC Institute of Urology and Catherine and Joseph Aresty Department of Urology, Keck School of Medicine, University of Southern California, Los Angeles; 2 Los Angeles Cancer Surveillance Program, Keck School of Medicine, University of Southern California, Los Angeles; 3 Keck School of Medicine, University of Southern California, Los Angeles; 4 Norris Comprehensive Cancer Center, Keck School of Medicine, University of Southern California, Los Angeles

## Abstract

**Importance:**

The US Preventive Services Task Force (USPSTF) has recommended against routine prostate-specific antigen (PSA)–based prostate cancer (PCa) screening, initially for men older than 75 years in 2008, and then for all men in 2012. Concern has been raised that, by recommending against screening, and thus early detection, the USPSTF recommendations may be associated with an increase in the incidence of metastatic PCa (mPCa).

**Objective:**

To explore the incidence of mPCa before and after the USPSTF recommendations against routine PCa screening.

**Design, Setting, and Participants:**

This population-based cohort study used the recently released Surveillance, Epidemiology, and End Results (SEER) 18 registry incidence data to identify men aged 45 years and older with a diagnosis of invasive PCa from 2004 through 2018. Data were analyzed from January 1, 2004 to December 31, 2018.

**Exposure:**

Outcomes were assessed before vs after the USPSTF recommendations against routine screening.

**Main Outcomes and Measures:**

Annual age-adjusted incidence rates per 100 000 population of mPCa (defined using SEER Summary Stage and American Joint Committee on Cancer [AJCC] staging systems), with adjustments for age structure and reporting delay from 2004 to 2011, according to race and age were examined. Annual percentage changes (APCs) were calculated to quantify changes in the annual incidence rates.

**Results:**

From 2004 to 2018, a total of 836 282 patients with PCa were recorded in the SEER database; 26 642 (56.5%) distant mPCa cases were reported in men aged 45 to 74 years, and 20 507 (43.5%) cases were reported in men aged 75 years or older. Among men aged 45 to 74 years, the incidence rate of distant mPCa (SEER Summary staging) remained stable during 2004 to 2010 (APC, −0.4%; 95% CI, −1.7% to 1.1%; *P* = .60), then increased significantly during 2010 to 2018 (APC, 5.3%; 95% CI, 4.5% to 6.0%; *P* < .001). In men aged 75 years or older, the incidence rate of distant mPCa decreased from 2004 to 2011 (APC, −1.5%; 95% CI, −3.0% to 0%; *P* = .046), and then increased from 2011 to 2018 (APC, 6.5%; 95% CI, 5.1% to 7.8%; *P* < .001). Similar trends were also seen for M1 mPCa defined per the AJCC staging system. These increased trends in mPCa incidence were particularly significant in non-Hispanic White men (2010-2018 APC, 6.9%; 95% CI, 5.4% to 8.4%; *P* < .001).

**Conclusions and Relevance:**

Analysis of the emerging trends from the most recently released SEER data set (2004-2018) suggests that the incidence rates of mPCa have increased significantly and coincide temporally with the USPSTF recommendations against PCa screening across races and age groups. These mPCa trends are associated with reported changes in screening practices following the USPSTF recommendations.

## Key Points

**Question:**

What are the incidence trends of metastatic prostate cancer (mPCa) before and after the US Preventive Services Task Force (USPSTF) recommendations against routine PCa screening?

**Findings:**

In this cohort study of 836 282 patients with PCa from the Surveillance, Epidemiology, and End Results (SEER) database, before the change in USPSTF recommendations, the incidence rate of mPCa was stable among men aged 45 to 74 years and decreasing among men older than 75 years. After the changed USPSTF recommendations, the incidence rate of mPCa increased in men of all ages.

**Meaning:**

This study suggests that the incidence of mPCA is increasing and might be temporally associated with changes in clinical policy and/or practice (eg, USPSTF guidelines), which may explain such rapid changes in cancer epidemiological trends.

## Teaser

This cohort study examines the incidence rate of prostate cancer in men aged 45 years and older to determine whether rates of cancer increased after US Preventive Services Task Force recommendations against routine screening.

## Introduction

The introduction of prostate-specific antigen (PSA) screening almost 3 decades ago was followed by a substantial reduction in the incidence of metastatic disease, as well as a reduction in prostate cancer (PCa)–specific mortality.^1^ Despite these observations, the value of PSA screening has remained controversial because of the risk of overdiagnosis and overtreatment of low-risk PCa, which may outweigh the mortality reduction resulting from screening for higher-risk disease. The US Preventive Services Task Force (USPSTF) recommended against routine PSA screening, initially for men older than 75 years in 2008, followed by a recommendation against screening for all men in 2012. These recommendations were made primarily by data from 2 large trials evaluating the impact of PSA-based PCa screening, The European Randomized study for Screening of Prostate Cancer and the Prostate, Lung, Colorectal, and Ovarian Screening Trial, which were published at the same time.^2,3^ The European Randomized study for Screening of Prostate Cancer showed a 20% overall risk reduction in PCa-specific mortality over a 16-year follow-up, whereas the Prostate, Lung, Colorectal, and Ovarian Screening trial did not show any difference even with extended follow-up.^3,4^ The USPSTF recommendations have resulted in a substantial reduction in PSA screening and the number of men diagnosed with PCa.^5,6,7,8,9^ The recently released American Cancer Statistics have also shown a steep drop in incidence rates of invasive PCa and a plateaued mortality rate.^10^ Several studies using different data sources have assessed the trends of metastatic PCa (mPCa) relative to the USPSTF recommendation against PCa screening.^1,7,11,12,13,14,15,16,17,18^

The Surveillance Epidemiology and End Result (SEER) database of 18 cancer registries provides population-level data on cancer incidence trends representing approximately 28% of the US population. Two prior studies^1,13^ looking at PCa trends using SEER registries through 2013 did indicate a significant increase in mPCa incidence rates in men older than 75 years from 2011 to 2012 that held steady in 2013, but no change in trends in men younger than 75 years. However, given the slow nature of PCa progression, the short interval between the 2008 and 2012 USPSTF recommendations vis-à-vis the publication of the 2013 SEER data may not have been sufficient to show definitive trends in the emerging mPCa incidence rates. As such, here, we examine the recently updated SEER data through 2018 to assess incidence rate trends of mPCa.

## Methods

### Data Sources

Using SEER 18 registry Incidence Data, 2000 to 2018, we identified invasive PCa cases diagnosed between January 1, 2004, and December 31, 2018, among men aged 45 years and older. The SEER 18 registry includes the Alaska Native Tumor Registry, Connecticut, Detroit, Atlanta, Greater Georgia and Rural Georgia, San Francisco-Oakland, San Jose-Monterey, Greater California, Hawaii, Iowa, Kentucky, Los Angeles, Louisiana, New Mexico, New Jersey, Seattle-Puget Sound, and Utah registries. Data were deidentified and publicly available under a data use agreement with the US National Cancer Institute. Thus, this study was deemed exempt from institutional review board approval according to the 2018 Revised Common Rule (45 CFR §46). This study was carried out according to the Strengthening the Reporting of Observational Studies in Epidemiology (STROBE) reporting guideline.^19^

### Case Definitions and Variables

Invasive PCa cases were identified by using *International Classification of Diseases for Oncology, Third Edition (ICD-O-3)* site code C61.9 (excluding histology codes of 9050-9055, 9140, and 9590-9992) and behavior code 3.

Race and ethnicity were categorized as non-Hispanic White, non-Hispanic Black, and Hispanic with any race. Race and ethnicity were determined from the database and were assessed in this study because different races and ethnicities have shown different screening patterns and PCa risk. Age at diagnosis was grouped into 45 to 74 years and 75 years or older. Age was dichotomized at 75 years in accordance with the metric used by the USPSTF for issuing their recommendations. Tumor stage at diagnosis was defined by both SEER summary stage^20^ and the American Joint Committee on Cancer (AJCC) TNM staging system.^21^ Summary stage groups were categorized as localized or distant, whereas AJCC stage groups were categorized as T1 to T2, T3 to T4, N0, N1, M0, and M1 in the analysis. Metastatic prostate cancer was defined as having a distant SEER summary stage (referred to as distant mPCa) or M1 AJCC stage (referred to as M1 mPCa). Our use of both SEER summary and AJCC staging systems was aimed at minimizing misclassification bias. Gleason score on needle core biopsy or transurethral resection of prostate was categorized into 2 through 6, 7, and 8 through 10. Median PSA value at the time of diagnosis was calculated for each year and expressed in nanograms per milliliter (a lab value expressed in micrograms per liter is equivalent to the same value expressed in nanograms per milliliter).

### Statistical Analysis

We used SEER*Stat version 8.3.8 (National Cancer Institute) to calculate the age-adjusted (2000 US standard population) incidence rates (per 100 000 population) with delay-adjustment by age, race, and cancer type. Following SEER conventions, only rates based on 16 or more cases are reported.^22^ The Joinpoint Regression Analysis software version 4.8.0.1 (National Cancer Institute) was used to calculate annual percentage changes (APCs) with 95% CIs to quantify the changes in trends in delay-adjusted age-adjusted incidence rates. The software selects the best fitting log-linear regression model to identify calendar years when the APCs changed substantially. A sensitivity analysis was performed by doing joinpoint regression using a predetermined cutoff at 2010 for all age and race categories (eTable 5 in the Supplement) to measure the association of the 2008 USPSTF task force recommendations with cancer rates. The comparability test compared whether 2 sets of trend data whose mean functions are represented by joinpoint regression are parallel. The tests of significance use a Monte Carlo Permutation method with significance level at .05. All statistical tests were 2-sided. SAS statistical software version 9.4 (SAS Institute) was used to calculate the median (IQR) for PSA value. Data were analyzed from January 1, 2004, to December 31, 2018.

## Results

### Overall Population

From 2004 through 2018, a total of 836 282 patients with PCa were recorded in the SEER database, of whom 576 816 (68.6%) were non-Hispanic White, 124 322 (14.8%) were non-Hispanic Black, and 76 989 (9.2%) were Hispanic. In men 45 to 75 years old, there was no statistically significant change in the incidence rate of mPCa from 2004 to 2010 followed by an approximately 41% increase from 2010 to 2018. For men aged 75 years and older, there was a significant decline in incidence of mPCa from 2004 through 2011 followed by a 43% increase. Between 2004 and 2018, the median (IQR) PSA level at the time of diagnosis increased from 6.3 (4.7-9.7) ng/mL to 7.0 (5.1-11.4) ng/mL in men aged 45 to 74 years, with the lowest value recorded in 2009 (5.9 [4.5-9.0] ng/mL), and from 10.0 (6.2-21.2) ng/mL to 11.4 (6.8-32) ng/mL in men 75 years or older, with the lowest value recorded in 2008 (9.0 [5.7-18.1] ng/mL) (Table). We noted rising incidence rates of N1 disease from 2004 to 2018 (119% increase and 81% increase in men aged ≥75 and 45-74, respectively) and T3 and T4 stage from 2013 to 2018 (38% increase and 31% increase in men aged ≥75 and 45-74, respectively).

**Table.** Yearly Variations in Prostate Cancer Presentation at Diagnosis: Surveillance Epidemiology and End Result 18 Database 2004-2018, All Races and Ethnicities

Group and characteristics	Year														
	2004	2005	2006	2007	2008	2009	2010	2011	2012	2013	2014	2015	2016	2017	2018
Men aged <75 y															
No. of patients	40 610	39 192	44 198	47 961	46 369	47 812	47 049	48 352	41 753	40 775	38 275	40 877	43 260	46 335	45 893
PSA level, median (IQR), ng/mL	6.3 (4.7-9.7)	6.2 (4.6-9.8)	6.1 (4.6-9.5)	6.0 (4.5-9.1)	6.0 (4.5-9)	5.9 (4.5-9.1)	6.0 (4.6-9.2)	6.0 (4.6-9.1)	6.2 (4.7-9.7)	6.5 (4.8-10.2)	6.7 (4.9-10.7)	6.9 (5-11.1)	6.9 (5-11.1)	7.0 (5.1-11.3)	7.0 (5.1-11.4)
Delay-adjusted incidence rates, cases/100 100 men															
Invasive PCa	378.67	356.49	390.79	409.25	380.01	377.52	359.3	358.45	298.73	282.58	257.42	267.31	276.52	291.82	288.91
Localized PCa	307.78	289.58	322.76	335.47	307.87	303.5	287.59	287.64	235.92	220.1	195.5	199.83	198.33	206.55	209.28
Localized plus regional	359.24	336.51	371.11	387.65	356.83	353.26	335.17	334.26	276.06	258.35	232.66	241.13	240.45	250.45	251.7
Localized or metastatic PCa	27.24	24.69	27.73	30.33	27.46	26.76	24.58	23.35	19.18	16.89	14.23	14.17	12.81	12.63	12.30
Metastasis^a^															
Summary stage (distant)	11.58	11.92	11.78	11.26	11.41	11.51	11.83	12.39	12.41	13.16	13.84	14.31	15.98	16.66	17.3
AJCC M1	11.3	11.73	11.64	11.06	11.21	11.34	11.7	12.32	12.3	13.03	13.74	14.1	15.48	16.35	17.02
AJCC N1	6.68	6.93	6.91	7.55	7.63	7.73	8.36	8.32	8.6	8.94	10.9	12.3	13.21	14.73	14.9
T stage^a^															
T1-T2	331.82	310.95	343.95	357.58	327.39	322	304.43	304.62	250.87	234.1	208.67	212.98	205.45	209.71	218.22
T3-T4	36.05	34.27	35.68	38.51	37.78	39.56	39.47	38.75	34.55	33.77	34.04	38.44	42.2	44.77	43.64
Gleason score^a^															
≤6	189.23	173.75	184.58	188.47	162.62	125.27	167.29	164.59	128.55	113.14	96.22	93.82	92.93	93.68	88.00
7	122.47	120.72	138.02	151.13	146.08	119.95	123.62	125.08	107.10	103.67	96.02	103.52	111.83	116.60	115.88
8-10	43.78	43.51	47.95	48.16	45.37	37.95	48.03	47.77	44.60	45.93	45.95	49.43	52.59	56.85	54.34
Men aged ≥75 y															
No. of patients	14 426	13 773	14 393	14 541	12 896	12 115	11 882	11 537	9763	9610	9246	9941	10 521	11 295	11 632
PSA level, median (IQR), ng/mL	10.0 (6.2-21.2)	9.7 (6.1-20)	9.4 (44366.0)	9.1 (5.8-17.9)	9.0 (5.7-18.1)	9.0 (5.7-18.8)	9.4 (5.9-20.1)	9.1 (5.8-19.5)	10.2 (6.2-25.9)	10.6 (6.4-28.7)	11.2 (6.6-31.65)	11.7 (6.9-33.2)	11.5 (6.8-32)	11.6 (6.9-31.7)	11.4 (6.8-32)
Delay-adjusted incidence rates, cases/100 100 men															
Invasive PCa	838.33	785.81	809.7	807.79	709.03	659.07	637.68	609.53	506.1	487.46	457.7	480.75	497.74	522.79	524.56
Localized PCa	657.99	610.38	638.92	625.51	531.09	480.56	458.98	453.17	349.6	325.66	295.22	304.18	304.35	313.5	325.64
Localized plus regional	689.74	639.51	669.94	657.46	561.27	510.77	492.51	482.85	378.02	352.54	323.7	337.89	342.71	353.84	367.76
Localized or metastatic PCa	9.91	9.42	9.74	10.09	8.87	8.12	7.57	7.85	5.31	4.74	4.25	3.79	3.83	3.59	3.69
Metastasis^a^															
Summary stage (distant)	67.26	65.57	66.52	63.1	60.91	59.84	61.21	58.14	66.18	69.08	70.09	80.77	80.72	90.53	88.97
AJCC M1	66.43	64.79	65.6	61.97	59.85	59.2	60.62	57.72	65.83	68.67	69.46	80.3	79.39	87.42	88.16
AJCC N1	9.12	9.81	10.71	13.12	13.15	13.75	14.68	15.93	17.97	19.1	22.47	25.62	29.15	32.28	34.63
T stage^a^															
T1-T2	689.96	643.1	671.72	659.95	562.04	509.67	490.29	484.49	382.32	359.01	326.97	340.48	325.27	337.42	363.33
T3-T4	41.5	37.67	38.55	37.2	36.34	35.48	39.15	33.06	34.04	34	37.24	42.89	48.22	50.37	50.86
Gleason score^a^															
≤6	289.07	263.89	265.95	256.59	208.95	137.70	162.01	158.97	110.90	105.91	82.79	77.91	82.80	82.30	78.50
7	237.62	227.08	239.15	252.33	221.47	147.29	197.78	195.40	145.37	133.98	122.63	135.16	143.00	148.99	146.75
8-10	171.50	164.00	178.56	173.88	151.74	113.30	162.64	155.89	138.89	135.46	139.97	149.79	163.53	171.28	171.99

Abbreviations: AJCC, American Joint Committee on Cancer; PCa, prostate cancer; PSA, prostate-specific antigen.

SI conversion factor: To convert PSA level to micrograms per liter, multiply by 1.

^a^ Rates are per 100 000 and age-adjusted to the 2000 US Standard Population standard.

### Trends for mPCa

A total of 26 642 (56.5%) and 20 507 (43.5%) distant mPCa cases were reported in men aged 45 to 74 and 75 years or older, respectively. Panel A in the Figure and eFigure 1 in the Supplement depict trends over time for the delay-adjusted incidence rate per 100 000 men for cases categorized as distant-stage according to the SEER summary stage by age groups (distant mPCa). Considering all races (Figure A and eTable 1 in the Supplement) in the population aged 45 to 74 years, the distant mPCa incidence rate remained stable between 2004 (11.58 cases per 100 000 men) and 2010 (11.83 cases per 100 000 men) (APC, −0.4%; 95% CI, −1.7% to 1.1%; *P* = .60), and then significantly increased through 2018 (17.30 cases per 100 000 men) (APC, 5.3%; 95% CI, 4.5% to 6.0%; *P* < .001). In men aged 75 years or older, the distant mPCa incidence rate per 100 000 men decreased significantly from 67.26 cases per 100 000 men in 2004 to 58.14 cases per 100 000 men in 2011 (APC, −1.5%; 95% CI, −3.0% to 0%; *P* = .046) and then increased significantly to 88.97 cases per 100 000 men in 2018 (APC, 6.5%; 95% CI, 5.1% to 7.8%; *P* < .001).

**Figure.**
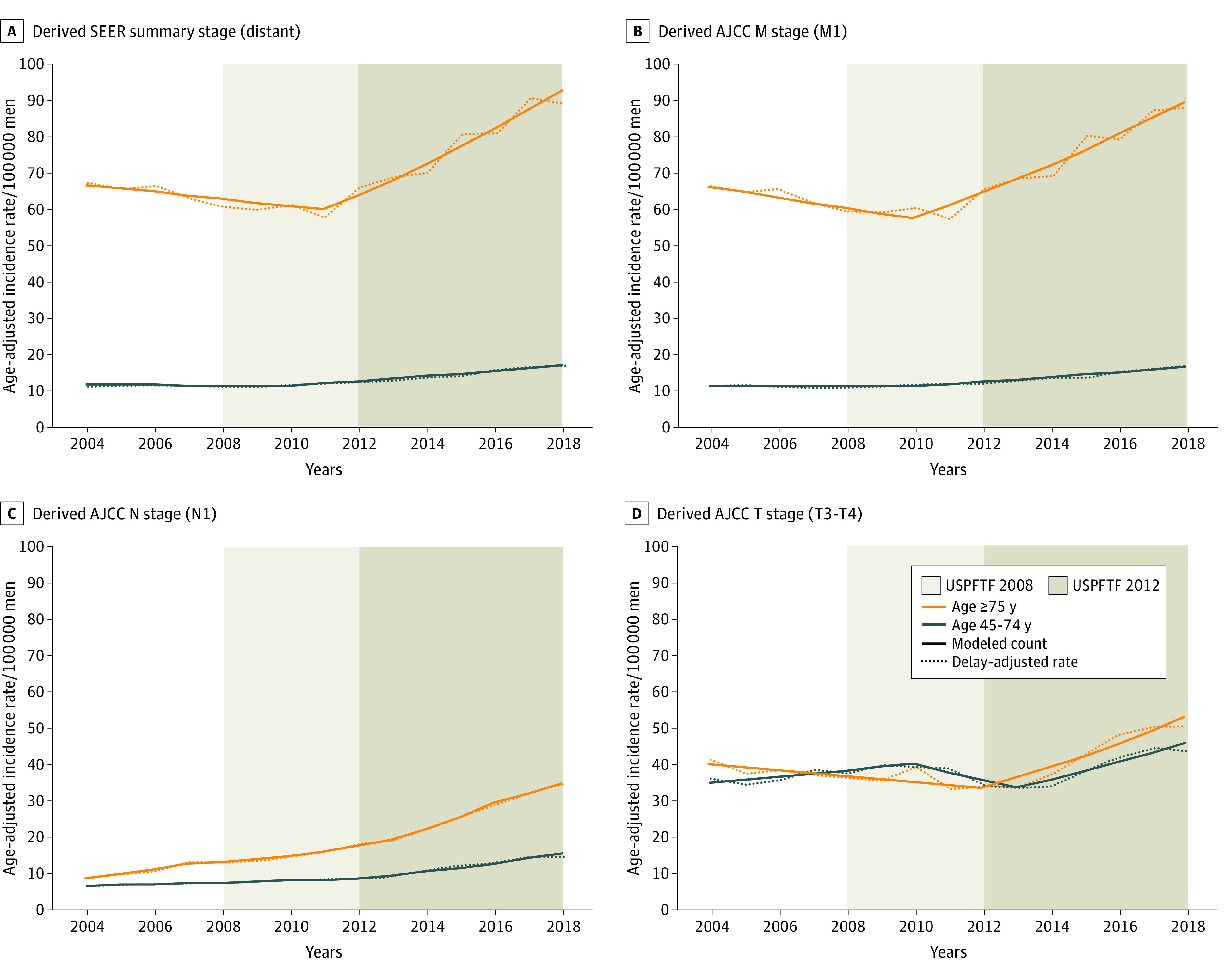
Trends in Invasive Prostate Cancer: Surveillance, Epidemiology, and End Results 18 Registries 2004-2018 for All Races The line segments of each curve were calculated with the Joinpoint Regression Analysis program. For each cohort, we compared the percentage difference in incidence rate (IR) between the lowest and the highest modeled value. Panel A shows data for derived SEER summary stage (distant); in men older than 75 years, the IR of metastatic prostate cancer (mPCa) increased by 43% from 2011 to 2018, from an annual percentage change (APC) of −1.5% to 6.5%, and in men aged 45 to 74, the IR of mPCa increased by 41% from 2010 to 2018, from an APC of −0.4% to 5.3%. Panel B shows data for derived American Joint Committee on Cancer (AJCC) M-stage (M1); in men older than 75 years, the IR of mPCa increased by 43% from 2010 to 2018, from an APC of −2.3% to 5.6%, and in men aged 45 to 74 years, the IR of mPCa increased by 39% from 2010 to 2018, from an APC of −0.1% to 5.1%. Panel C shows data for derived AJCC N-stage (N1); in men older than 75 years, the IR of N1 disease increased by 119% from 2004 to 2018, from an APC of 12.6% to 8.8%, and in men aged 45 to 74 years, the IR of N1 disease increased by 81% from 2004 to 2018, from an APC of 3.4% to 10.4%. Panel D shows data for derived AJCC T-stage (T3-T4); in men older than 75 years, the IR of T3-T4 disease increased by 45% from 2012 to 2018, from an APC of 2.3% to 7.9%, and in men aged 45 to 74 years, the IR of T3-T4 disease increased by 31% from 2013 to 2018, from an APC of −2.2% to 6.5%. Trends categorized by race are reported in eTable 1, eTable 2, and eTable 3 in the Supplement.

Considering only the non-Hispanic White population (eTable 1, eTable 4, eFigure 1, eFigure 2, eFigure 3, and eFigure 4 in the Supplement) in the population aged 45 to 74 years, the distant mPCa incidence rate remained stable between 2004 (9.66 cases per 100 000 men) and 2012 (10.7 cases per 100 000 men) (APC, 1.4%; 95% CI, 0% to 2.8%; *P* = .047), and then significantly increased through 2018 (15.88 cases per 100 000 men) (APC, 7.5%; 95% CI, 5.7% to 9.3%; *P* < .001). In men aged 75 years or older, the distant mPCa incidence rate per 100 000 men decreased from 60.27 cases per 100 000 men in 2004 to 57.88 cases per 100 000 men in 2010, although the decrease was not significant (APC, −1.7%; 95% CI, −4.2% to −0.8%; *P* = .16) and then increased significantly to 92.66 cases per 100 000 men in 2018 (APC, 6.9%; 95% CI, 5.4% to 8.4%; *P* < .001).

Considering the non-Hispanic Black population (eTable 3, eTable 5, eFigure 1, eFigure 2, eFigure 3, and eFigure 4 in the Supplement) in the population aged 45 to 74, the distant mPCa incidence rate remained stable between 2004 (27.02 cases per 100 000 men) and 2012 (29.43 cases per 100 000 men) (APC, 0.5%; 95% CI, −1.3% to 2.3%; *P* = .60), and then significantly increased through 2018 (39.68 cases per 100 000 men) (APC, 5.0%; 95% CI, 2.7% to 7.3%; *P* < .001). In men aged 75 years or older, the distant mPCa incidence rate per 100 000 men decreased significantly from 143.94 cases per 100 000 men in 2004 to 96.92 cases per 100 000 men in 2012 (APC, −3.7%; 95% CI, −6.7% to −0.5%; *P* = .03) and then increased significantly to 125.81 cases per 100 000 men in 2018 (APC, 6.2%; 95% CI, 1.6% to 11.1%; *P* = .01).

Considering the Hispanic population (eTable 3, eTable 4, eFigure 1, eFigure 2, eFigure 3, and eFigure 4 in the Supplement) in the population aged 45 to 74 years, the distant mPCa incidence rate increased significantly from 2004 (13.64 cases per 100 000 men) to 2018 (15.71 cases per 100 000 men) (APC, 1.3%; 95% CI, −0.3% to 2.3%; *P* = .01). In men aged 75 years or older, the distant mPCa incidence rate per 100 000 men decreased from 91.53 cases per 100 000 men in 2004 to 61.98 cases per 100 000 men in 2006 (APC, −20.6%; 95% CI, −44% to −12.4%; *P* = .17) and then increased significantly to 90.49 cases per 100 000 men in 2018 (APC, 3.0%; 95% CI, 1.2% to 4.8%; *P* = .004). Similar trends have been confirmed for age-adjusted incidence rate trends for mPCa based on M1 AJCC stage (eTable 4 in the Supplement).

In our sensitivity analysis, we tested the joinpoint regression analysis of trends over time for the delay-adjusted age-adjusted incidence rates per 100 000 men for mPCa cases categorized by age groups and race by fixing the time periods 2004 to 2010 and 2010 to 2018 (eTable 5 in the Supplement). For distant PCa among all races, during the 2004 to 2010 period, the delay-adjusted incidence rates remained stable (APC, −0.1%; 95% CI, 1.2% to 1.0%; *P* = .80) for men aged 45 to 74 years, and the test of parallelism was significantly rejected (*P* = .02) when compared with the trend of incidence in men aged 75 years or older, which declined significantly (APC, −2%; 95% CI,-2.9% to -0.9; *P* = .004). However, during the period 2010 to 2018, incidence rates of both age groups 45 to 74 years and 75 years or older increased significantly (APC, 5.2%; 95% CI, 4.4% to 6.0% *P* < .001 vs APC, 5.8%; *P* < .001, respectively) showing similar rising trends (*P* = .43). The trend of incidence in derived AJCC M1 PCa showed a similar pattern as distant PCa for both age groups in all races during 2 time periods, and their parallelism test results were similar as well (eTable 5 in the Supplement).

## Discussion

In this cohort study of the recently released 2004 to 2018 SEER registry data set, we found an overall increase in the incidence rates of mPCa since the USPSTF recommendations of 2008 and 2012,^2,3^ despite a significant reduction in the overall incidence of PCa diagnosis during this same period. This stands in contrast to the decreasing trends in incidence of mPCa between 2004 to 2009, preceding the USPSTF recommendations. Although the reasons behind this recent rising incidence of mPCa are multifactorial, it is unlikely to be due to a true change in cancer biology in such a short period. Factors such as environmental exposures or germline variations leading to changes in epidemiological signatures of cancers take substantially longer. Rather, changes in clinical policy and/or practice such as screening strategies and use of diagnostic imaging are much more likely to explain such short-term changes in cancer epidemiological trends.^23,24,25^ The introduction and rapid adoption of PSA in the early 1990s was associated with a major increase in PCa incidence.^24^ The negative consequences of such early detection were overdiagnosis and overtreatment^24^; its purported positive consequences were a reduction in the incidence of metastatic disease and almost 50% reduction in mortality over the next decade.^23,25^

Although the SEER data provide a representative sample of the adult US population to study cancer trends, they lack granular details, specifically data on screening practices. Therefore, we studied the chronological trend analysis of mPCa vis-à-vis the timing of USPSTF recommendations to assess for any plausible associations. In men 45 to 75 years old, there was a nonsignificant decline in the incidence rate of mPCa from 2004 to 2010 followed by an approximately 41% increase from 2010 to 2018. For men aged 75 years and older, there was a significant decline in incidence of mPCa from 2004 through 2011 followed by an even steeper 43% increase (Figure). It is important to reconcile the chronology of the increased mPCa trends relative to the USPSTF recommendations specifically in terms of lead time of diagnosis as a result of screening and the time to progression from localized to metastatic disease. The lead time for diagnosis of PCa in the screening group compared with the control group was 3.4 years and 1.5 years in the ESRPC^2^ and Prostate, Lung, Colorectal, and Ovarian trials, respectively. In the active monitoring group of the Prostate Testing for Cancer and Treatment trial, comprising a reasonably low-risk cohort, 6% of patients progressed to metastatic disease within a 10-year follow-up; of note, time to progression to metastatic disease is not available in the published Prostate Testing for Cancer and Treatment trial data.^26^ The observation arm of the Prostate Cancer Intervention Versus Observation Trial presents a more representative sample of risk distribution of PCa in the US population, wherein diagnosis of metastatic disease, based primarily on periodic bone imaging, was observed in 15% patients in the Prostate Cancer Intervention Versus Observation Trial starting at around the 3-year mark from randomization. Finally, when looking at the 1988 to 2012 SEER data, the reduction in incidence of mPCa similarly started approximately 3 years after introduction of PSA screening.^25^

The differences in trends between the age groups (45-74 vs ≥75 years) also follows the pattern of change in PSA screening practices after the USPSTF recommendations came into effect. Several studies have evaluated the impact of USPSTF recommendations on PSA screening practices, using either cross-sectional population survey data^7,27,28^ or individual health system claims data.^5,6,8,9^ Assessment of the true impact of screening practices is limited by recall bias with the cross-sectional survey data, as well as misclassification bias and low generalizability with individual health system claims data. Despite these limitations, several observations emerge from these studies. Overall, PSA screening decreased after the USPSTF recommendations against screening, with the greatest decline following the 2012 update.^8^ Although most studies have shown that reduction in PSA screening extended to all age groups, there is some evidence that there was an earlier and greater reduction in screening in men older than 75 years.^5^ Data from the National Health Information Survey on PSA screening trends following the 2008 USPSTF recommendation indicated that compared with 2008, PSA screening decreased in 2010 by 6% and 14% for men younger than 75 and older than 75 years, respectively; from 2010 through 2012, this reduction was 19% and 16%, respectively.^4^ This reduction in PSA screening occurred contemporaneously with our observations on the rising incidence of mPCa. Although the incidence of mPCa increased in both age groups, the rise in men aged 75 years and older was steeper (APC 6.5% vs 5.3%). Additionally, the first year-to-year significant rise in the incidence rate of mPCa in men older than 75 was observed between the years 2011 and 2012, compared with 2015 and 2016 for younger men. The rise in incidence of mPCa in men younger than 75 predominantly occurred in the latter years (2015-2018) of the SEER data set. Since the recommendation against screening was made 4 years earlier for older men, these differences in temporal trends between the age groups also suggest a likely association. Two previous studies^1,13^ looking at SEER 18 registry data through 2013 also showed a significant increase in the incidence rate and proportion of men older than 75 presenting with metastatic disease between the years 2011 and 2012, but no increase in younger men. More recently, Jemal et al^16^ evaluated the trends in newly diagnosed localized, regional, and metastatic PCa between 2005 and 2016 using the US Cancer Statistics Public Use Research Database, which merges SEER and US Centers for Disease Control and Prevention data to provide more comprehensive data about cancer epidemiology in the US than either source alone. They also found a significant drop in incidence rates of localized disease with a contrasting increased incidence rate in regional and metastatic disease. This study was limited by lack of delay adjustment. To our knowledge, the present analysis is the first to evaluate the trend through 2018 and also adjusts for delay in reporting.^16^

To minimize misclassification errors, we evaluated both the SEER summary-stage (distant) as well as AJCC SEER collaborative stage (M1) and found an increase in mPCa incidence rates in both. In addition to an absolute increase in incidence rates of mPCa, we found a similar rise in the proportion of metastatic vs localized disease.

Racial differences in mPCa trends appear to follow racial differences in PSA-based screening. In non-Hispanic-White men, there was little change in the incidence of mPCa from 2004 through 2012 (prior to the USPSTF recommendations), which was followed by a steep rise through 2018, regardless of age. A similar trend was seen in non-Hispanic b Black men older than 75 years who had a significant increase in mPCa after 2012. Non-Hispanic White and older Black men have higher PSA screening rates compared with other populations and are more likely to be impacted by changes in screening practices.^5,6^ Black men younger than 75 years showed a gradual yet persistent rise in mPCa incidence throughout the study period (2004-2018). Younger Black men have lower rates of PSA screening and so are less likely to be impacted by changes in screening practices.^6^

Since SEER lacks data that can demonstrate causality, alternative practice pattern causes should also be explored. Incorporating newer diagnostic and staging imaging techniques with higher sensitivity may also be associated with increased detection of low-volume metastatic disease and may be associated with the increased incidence of mPCa at diagnosis. Recent advances in molecular positron emission tomographic imaging using novel PCa-specific agents, such as fluciclovine and prostate-specific membrane antigen, offer increased sensitivity in detecting occult metastatic disease. However, this is unlikely to have been a major factor associated with the increase in mPCa in our study for several reasons. First, fluciclovine was approved for use in the US only in 2017,^29^ much later than our observed rise in metastatic disease. Second, fluciclovine and other molecular positron emission tomography agents have been approved for the setting of biochemical recurrence after definitive local therapy or monitoring of response of metastatic disease to systemic therapy, and it is therefore unlikely to impact the incidence of de novo metastatic disease at presentation. Finally, there is evidence that use of fluorine-18 fluciclovine and prostate-specific membrane antigen is not yet widespread.^30^ Going forward, the expected increased use of these imaging modalities, as well as the anticipated inclusion of prostate-specific membrane antigen-based positron emission tomographic imaging, will likely further be associated with the incidence rates of metastatic and locally advanced PCa.

If indeed this increased incidence rate of mPCa is associated with reduced PSA screening, it has important implications for overall PCa morbidity and mortality. There is evidence of a recent rise in incidence rates^16,31^ of higher grade and stage at diagnosis, coincident with USPSTF recommendations.^11^ Even in the present study, which provides the most updated data available, we noted rising incidence rates of N1 disease from 2004 to 2018 (119% increase and 81% increase in men aged ≥75 and 45-74, respectively) and T3 or T4 stage from 2013 through 2018 (38% increase and 31% increase in men aged ≥75 and 45-74, respectively) when comparing time periods before and after the USPSTF recommendations. It is likely that this may eventually translate into a higher future incidence of mPCa and PCa mortality. If the increased metastatic cases are indeed a result of reduced screening, this latent patient pool may represent an even greater disease burden compared with men with screen-detected metastatic disease.

Interestingly, trends similar to the US have been reported from other countries that followed USPSTF recommendations. In an Australian study,^32^ the incidence of newly diagnosed mPCa before and after USPSTF recommendations was 17.7% and 31.5%, respectively (*P* < .05). However, opposite trends were reported in countries where USPSTF recommendations have not been followed. The European Randomized Study of Screening for Prostate Cancer^33^ reported a 1.6-fold increase in PCa incidence and 21% reduction in PCa mortality in a PSA screening-based program. Comparing mPCa incidence rate ratios for screening vs control groups by risk category showed a reduction in metastatic disease at diagnosis in the screening arm (incidence rate ratio, 0.60; 95% CI, 0.52-0.70), preceding mortality reduction by 3 years.^33^

Regardless of the cause, the observation of a rising incidence of mPCa in itself does not imply that screening practices should be changed. The overall risk vs benefit of PSA-based screening is extremely complex and must take into account various other factors that impact the overall health of the community. As such, this is the first time that a sustained increase in the incidence rate of mPCa in all age and race groups has been shown using SEER data that we know of. We believe this is an important observation that merits further evaluation.

### Limitations

Our study has several limitations. First, SEER lacks granular data elements, including screening data as well as data on diagnostic techniques used, and therefore is unable to definitively determine causality of epidemiological signatures. SEER is considered to represent approximately 28% of the population. Second, the most recent SEER data set is through 2018 and therefore trends beyond that are not yet available. This is important because given PCa’s slow disease biology, longer follow-up is important to get a fuller picture on various trends. Because of the approximately 2-year delay in the release of the complete, population-based SEER registry data, an earlier, interim signal about incidence trends of mPCa and PCa-specific mortality could potentially be obtained by analysis of real-time search engine infodemiological data.^34^ Third, in 2018 USPSTF modified its recommendations to a category C from its prior category D in 2012, recommending shared decision-making of the pros and cons of PSA-based screening in men aged 55 to 70 years.^35^ The precise association of this change in screening patterns with epidemiological signatures of stage-specific and age-specific PCa remains unknown. Fourth, since SEER registries do not monitor disease progression or recurrence, patients recorded as having localized disease at presentation but whose cancer subsequently become metastatic are not captured. Thus, the cumulative incidence of mPCa in the population is unknown. In addition, given the 5 to 7 years median survival of men with mPCa, it is too early to see trends in PCa mortality.

## Conclusions

Our study of the recently released 2004 to 2018 SEER data set confirms a rising incidence rate of mPCa coinciding with the 2008 and 2012 USPSTF recommendations against PSA-based PCa screening. Although this increase was seen across all age groups, it was greater in men aged 75 years and older as well as in non-Hispanic White men and followed trends in PSA screening reduction. The increased mPCa incidence rates occurred despite a significant concurrent reduction in the overall incidence of PCa diagnosis. Since SEER cannot provide data to assess causality, this trend should be carefully studied further to see if it continues beyond 2018, and whether it is associated with a similar rise in PCa mortality.

## Notes

Supplement. **eTable 1. **Yearly Variations in Prostate Cancer Presentation at Diagnosis—SEER Database 2004-2018: Non-Hispanic White **eTable 2. **Yearly Variations in Prostate Cancer Presentation at Diagnosis—SEER Database 2004-2018: Non-Hispanic Black **eTable 3. **Yearly Variations in Prostate Cancer Presentation at Diagnosis—SEER Database 2004-2018: Hispanic **eTable 4. **Age-Adjusted Metastatic Prostate Cancer Rates and Joinpoint Analysis (SEER 18, 2004-2018) **eTable 5. **Age-Adjusted Metastatic Prostate Cancer Rates and Joinpoint Analysis (SEER 18, 2004-2018) **eFigure 1. **Trends in Malignant Prostate Cancer, SEER 18 Registries 2004-2018: Derived SEER summary stage (distant) by Race **eFigure 2. **Trends in Malignant Prostate Cancer, SEER 18 Registries 2004-2018: Derived AJCC M stage (M1) by Race **eFigure 3. **Trends in Malignant Prostate Cancer, SEER 18 Registries 2004-2018: Derived AJCC N stage (N1) by Race **eFigure 4. **Trends in Malignant Prostate Cancer, SEER 18 Registries 2004-2018: Derived AJCC T stage (T3-T4) by Race

## References

[1] Hu JC, Nguyen P, Mao J, . Increase in prostate cancer distant metastases at diagnosis in the United States. JAMA Oncol. 2017;3(5):705-707. doi:10.1001/jamaoncol.2016.5465 28033446 PMC5470389

[2] Hugosson J, Roobol MJ, Månsson M, ; ERSPC investigators. A 16-yr follow-up of the European Randomized study of Screening for Prostate Cancer. Eur Urol. 2019;76(1):43-51. doi:10.1016/j.eururo.2019.02.009 30824296 PMC7513694

[3] Pinsky PF, Prorok PC, Yu K, . Extended mortality results for prostate cancer screening in the PLCO trial with median follow-up of 15 years. Cancer. 2017;123(4):592-599. doi:10.1002/cncr.30474 27911486 PMC5725951

[4] Pinsky PF, Miller E, Prorok P, Grubb R, Crawford ED, Andriole G. Extended follow-up for prostate cancer incidence and mortality among participants in the prostate, lung, colorectal and ovarian randomized cancer screening trial. BJU Int. 2019;123(5):854-860. doi:10.1111/bju.14580 30288918 PMC6450783

[5] Cohn JA, Wang CE, Lakeman JC, . Primary care physician PSA screening practices before and after the final U.S. Preventive Services Task Force recommendation. Urol Oncol. 2014;32(1):41.e23-41.e30. doi:10.1016/j.urolonc.2013.04.013 23911680

[6] Frendl D, Epstein M, Fouayzi H, . MP39-06 impact of guidelines on prostate cancer screening in a population-based setting, 2000-2014: preliminary results from the first AUA data grant. J Urol. 2016;195(4S):e543. doi:10.1016/j.juro.2016.02.131

[7] Jemal A, Fedewa SA, Ma J, . Prostate cancer incidence and PSA testing patterns in relation to USPSTF screening recommendations. JAMA. 2015;314(19):2054-2061. doi:10.1001/jama.2015.14905 26575061

[8] Kearns JT, Holt SK, Wright JL, Lin DW, Lange PH, Gore JL. PSA screening, prostate biopsy, and treatment of prostate cancer in the years surrounding the USPSTF recommendation against prostate cancer screening. Cancer. 2018;124(13):2733-2739. doi:10.1002/cncr.31337 29781117

[9] Kim SP, Karnes RJ, Nguyen PL, . A national survey of radiation oncologists and urologists on recommendations of prostate-specific antigen screening for prostate cancer. BJU Int. 2014;113(5b):E106-E111. doi:10.1111/bju.12422 24053213 PMC4353641

[10] Siegel RL, Miller KD, Fuchs HE, Jemal A. Cancer statistics, 2022. CA Cancer J Clin. 2022;72(1):7-33. doi:10.3322/caac.21708 35020204

[11] Ahlering T, Huynh LM, Kaler KS, . Unintended consequences of decreased PSA-based prostate cancer screening. World J Urol. 2019;37(3):489-496. doi:10.1007/s00345-018-2407-3 30003374

[12] Dall’Era MA, deVere-White R, Rodriguez D, Cress R. Changing incidence of metastatic prostate cancer by race and age, 1988-2015. Eur Urol Focus. 2019;5(6):1014-1021. doi:10.1016/j.euf.2018.04.016 29735368

[13] Jemal A, Ma J, Siegel R, Fedewa S, Brawley O, Ward EM. Prostate cancer incidence rates 2 years after the US Preventive Services Task Force recommendations against screening. JAMA Oncol. 2016;2(12):1657-1660. doi:10.1001/jamaoncol.2016.2667 27541955

[14] Kelly SP, Anderson WF, Rosenberg PS, Cook MB. Past, current, and future incidence rates and burden of metastatic prostate cancer in the United States. Eur Urol Focus. 2018;4(1):121-127. doi:10.1016/j.euf.2017.10.014 29162421 PMC6217835

[15] Weiner AB, Matulewicz RS, Tosoian JJ, Feinglass JM, Schaeffer EM. The effect of socioeconomic status, race, and insurance type on newly diagnosed metastatic prostate cancer in the United States (2004-2013). Urol Oncol. 2018;36(3):91.e1-91.e6. doi:10.1016/j.urolonc.2017.10.023 29153624 PMC6048442

[16] Jemal A, Culp MB, Ma J, Islami F, Fedewa SA. Prostate cancer incidence 5 years after US preventive services task force recommendations against screening. J Natl Cancer Inst. 2021;113(1):64-71. doi:10.1093/jnci/djaa068 32432713 PMC7781461

[17] Kensler KH, Pernar CH, Mahal BA, . Racial and ethnic variation in PSA testing and prostate cancer incidence following the 2012 USPSTF recommendation. J Natl Cancer Inst. 2021;113(6):719-726. doi:10.1093/jnci/djaa171 33146392 PMC8168268

[18] Butler SS, Muralidhar V, Zhao SG, . Prostate cancer incidence across stage, NCCN risk groups, and age before and after USPSTF Grade D recommendations against prostate-specific antigen screening in 2012. Cancer. 2020;126(4):717-724. doi:10.1002/cncr.32604 31794057

[19] von Elm E, Altman DG, Egger M, Pocock SJ, Gøtzsche PC, Vandenbroucke JP; STROBE Initiative. The Strengthening the Reporting of Observational Studies in Epidemiology (STROBE) statement: guidelines for reporting observational studies. Bull World Health Organ. 2007;85(11):867-872. doi:10.2471/BLT.07.045120 18038077 PMC2636253

[20] Ruhl J, Hurlbut, A, Ries LAG, Adamo P, Dickie L, Schussler N. Summary Stage 2018: Codes and Coding Instructions. National Cancer Institute; 2020.

[21] Amin MB, Greene FL, Edge SB, . The Eighth Edition AJCC Cancer Staging Manual: continuing to build a bridge from a population-based to a more “personalized” approach to cancer staging. CA Cancer J Clin. 2017;67(2):93-99. doi:10.3322/caac.21388 28094848

[22] National Cancer Institute and US Centers for Disease Control and Prevention. Suppression: state cancer profiles. 2020. Accessed February 1, 2022. https://statecancerprofiles.cancer.gov/suppressed.html

[23] Welch HG, Kramer BS, Black WC. Epidemiologic signatures in cancer. N Engl J Med. 2019;381(14):1378-1386. doi:10.1056/NEJMsr1905447 31577882

[24] Welch HG, Albertsen PC. Prostate cancer diagnosis and treatment after the introduction of prostate-specific antigen screening: 1986-2005. J Natl Cancer Inst. 2009;101(19):1325-1329. doi:10.1093/jnci/djp278 19720969 PMC2758309

[25] Welch HG, Gorski DH, Albertsen PC. Trends in metastatic breast and prostate cancer: lessons in cancer dynamics. N Engl J Med. 2015;373(18):1685-1687. doi:10.1056/NEJMp1510443 26510017

[26] Hamdy FC, Donovan JL, Lane JA, ; ProtecT Study Group. 10-Year outcomes after monitoring, surgery, or radiotherapy for localized prostate cancer. N Engl J Med. 2016;375(15):1415-1424. doi:10.1056/NEJMoa1606220 27626136

[27] Abdollah F, Dalela D, Sood A, . PD15-01 The impact of 2012 United States Preventive Services Task Force (USPSTF) panel update on PSA screening practice: a nationwide, and state-by-state level analyses. J Urol. 2016;195(4S):e388-e389. doi:10.1016/j.juro.2016.02.1127

[28] Li J, Berkowitz Z, Hall IJ. Decrease in prostate cancer testing following the US Preventive Services Task Force (USPSTF) Recommendations. J Am Board Fam Med. 2015;28(4):491-493. doi:10.3122/jabfm.2015.04.150062 26152440 PMC6077843

[29] Blue Earth Diagnostics. Axumin prescribing information. 2016. Accessed February 1, 2022. https://www.accessdata.fda.gov/drugsatfda_docs/label/2016/208054s000lbl.pdf

[30] Li R, Ravizzini GC, Gorin MA, . The use of PET/CT in prostate cancer. Prostate Cancer Prostatic Dis. 2018;21(1):4-21. doi:10.1038/s41391-017-0007-8 29230009

[31] Iyer HS, Gomez SL, Chen JT, Trinh QD, Rebbeck TR. Trends in mortality among Black and White men with prostate cancer in Massachusetts and Pennsylvania: race and neighborhood socioeconomic position. Cancer. 2021;127(14):2525-2534. doi:10.1002/cncr.33506 33798264 PMC8249310

[32] Smith S, Wolanski P. Metastatic prostate cancer incidence in Australia after amendment to prostate-specific antigen screening guidelines. ANZ J Surg. 2018;88(7-8):E589-E593. doi:10.1111/ans.14275 29194902

[33] Buzzoni C, Auvinen A, Roobol MJ, . Metastatic prostate cancer incidence and prostate-specific antigen testing: new insights from the European randomized study of screening for prostate cancer. Eur Urol. 2015;68(5):885-890. doi:10.1016/j.eururo.2015.02.042 25791513 PMC4982869

[34] Cacciamani GE, Gill K, Gill IS. Web search queries and prostate cancer. Lancet Oncol. 2020;21(4):494-496. doi:10.1016/S1470-2045(20)30138-8 32950120

[35] Grossman DC, Curry SJ, Owens DK, ; US Preventive Services Task Force. Screening for prostate cancer: US Preventive Services Task Force recommendation statement. JAMA. 2018;319(18):1901-1913. doi:10.1001/jama.2018.3710 29801017

