# Supplementary material for: Trends in Incidence of Metastatic Prostate Cancer in the US
Source: JAMA Netw Open. 2022 Mar 14;5(3):e222246. doi: 10.1001/jamanetworkopen.2022.2246 (PMC9907338; doi:10.1001/jamanetworkopen.2022.2246)
Supplement: Supplement. — eTable 1. Yearly Variations in Prostate Cancer Presentation at Diagnosis—SEER Database 2004-2018: Non-Hispanic White eTable 2. Yearly Variations in Prostate Cancer Presentation at Diagnosis—SEER Database 2004-2018: Non-Hispanic Black eTable 3. Yearly Variations in Prostate Cancer Presentation at Diagnosis—SEER Database 2004-2018: Hispanic eTable 4. Age-Adjusted Metastatic Prostate Cancer Rates and Joinpoint Analysis (SEER 18, 2004-2018) eTable 5. Age-Adjusted Metastatic Prostate Cancer Rates and Joinpoint Analysis (SEER 18, 2004-2018) eFigure 1. Trends in Malignant Prostate Cancer, SEER 18 Registries 2004-2018: Derived SEER summary stage (distant) by Race eFigure 2. Trends in Malignant Prostate Cancer, SEER 18 Registries 2004-2018: Derived AJCC M stage (M1) by Race eFigure 3. Trends in Malignant Prostate Cancer, SEER 18 Registries 2004-2018: Derived AJCC N stage (N1) by Race eFigure 4. Trends in Malignant Prostate Cancer, SEER 18 Registries 2004-2018: Derived AJCC T stage (T3-T4) by Race [file jamanetwopen-e222246-s001.pdf]

Supplemental Online Content

Desai MM, Cacciamani G, Gill K, et al. Trends in the incidence of metastatic prostate cancer in the US. *JAMA Netw Open*. 2022;5(3):e222246. doi:10.1001/jamanetworkopen.2022.2246

eTable 1. Yearly Variations in Prostate Cancer Presentation at Diagnosis - SEER Database 2004-2018- Non-Hispanic White.
eTable 2. Yearly Variations in Prostate Cancer Presentation at Diagnosis - SEER Database 2004-2018- Non-Hispanic Black.
eTable 3. Yearly Variations in Prostate Cancer Presentation at Diagnosis - SEER Database 2004-2018- Hispanic.
eTable 4. Age-Adjusted metastatic Prostate Cancer rates and Joinpoint analysis (SEER 18, 2004-2018).
eTable 5. Age-Adjusted metastatic Prostate Cancer rates and Joinpoint analysis (SEER 18, 2004-2018). Joinpoint analysis modeled for periods 2004-2010 and 2010-2018.
eFigure 1. Trends in malignant prostate cancer, SEER 18 Registries 2004-2018. Derived SEER summary stage (distant) by race.
eFigure 2. Trends in malignant prostate cancer, SEER 18 Registries 2004-2018. Derived AJCC M stage (M1) by race.
eFigure 3. Trends in malignant prostate cancer, SEER 18 Registries 2004-2018. Derived AJCC N stage (N1) by race.
eFigure 4. Trends in malignant prostate cancer, SEER 18 Registries 2004-2018. Derived AJCC T stage (T3-T4) by race.

This supplemental material has been provided by the authors to give readers additional information about their work.

© 2022 Desai MM et al. *JAMA Network Open.*

SUPPLEMENTARY MATERIALS

INCIDENCE OF METASTATIC PROSTATE CANCER IN UNITED STATES CONTINUES TO INCREASE

Mihir M Desai[1*‡] MD, MPH, Giovanni Cacciamani[1,‡], MSc, MD, Juanjuan Zhang[2], Lihua Liu[2], Ph.D, Karanvir Gill[1], Andre Abreu[1], MD, Inderbir S Gill[1], MD, MCh

[1] USC Institute of Urology and Catherine and Joseph Aresty Department of Urology, Keck School of Medicine, University of Southern California, Los Angeles, California *and* [2] Los Angeles Cancer Surveillance Program, Keck School of Medicine, University of Southern California; Keck School of Medicine, University of Southern California, Los Angeles, California

© 2022 Desai MM et al. *JAMA Network Open.*

Supplementary tables

eTable 1. *Yearly Variations in Prostate Cancer Presentation at Diagnosis - SEER Database 2004-2018- Non-Hispanic White. * Rates are per 100,000 and age-adjusted to the 2000 US Std Population standard*

Delay-Adjusted Prostate Cancer Incidence (per 100 000 Men) in men < 75 old

	2004	2005	2006	2007	2008	2009	2010	2011	2012	2013	2014	2015	2016	2017	2018
No. of patients	28,836	27,548	31,442	33,924	32,454	32,899	32,159	32,928	27,673	26,932	25,085	26,508	27,992	29,511	28,979
PSA, ng/mL, median (IQR)	6.0	6.0	5.9	5.8	5.7	5.7	5.8	5.8	6.0	6.2	6.4	6.6	6.7	6.7	6.8
	4.6-9	4.5-9	4.5-8.8	4.4-8.5	4.4-8.4	4.4-8.4	4.5-8.5	4.4-8.5	4.6-9	4.7-9.5	4.7-9.9	4.9-10.2	4.9-10.3	5-10.5	5-10.5
Incidence rate Invasive PCa	372.68	350.3	391.34	409.76	379.29	372.2	355.06	355.81	290.56	276.1	252.54	261.8	273.05	287.2	287.84
Incidence rate Localized PCa	303.9	285.6	323.54	338.42	309.15	301.87	286.13	287.67	230.65	216.95	193.9	197.47	198.95	207.91	213.22
Incidence rate Localized + regional	356.18	333.72	374.38	392.93	360.52	353.46	335.69	336.49	272.71	257.1	232.93	240.81	244.25	255.31	259.75
Localized/metastatic PCa	32.16	29.29	32.81	36.86	32.44	32.11	28.39	26.42	21.82	19.49	15.96	15.60	14.31	13.56	13.67
Metastasis *															
Summary Stage (distant)	9.66	9.85	10.02	9.35	9.76	9.54	10.18	10.94	10.7	11.28	12.22	12.85	14.38	15.59	15.88
AJCC M1	9.45	9.75	9.86	9.18	9.53	9.4	10.08	10.89	10.57	11.13	12.15	12.66	13.9	15.33	15.6
AJCC N1	6.54	6.67	6.49	7.18	7.48	7.29	7.83	8.25	8.13	8.59	10.3	12	12.77	14.63	14.91
T Stage *															
T1-T2	326.91	306.39	344.83	360.29	328.29	319.65	302.31	304.41	245.12	230.3	206.32	209.82	205.31	211.98	221.81
T3-T4	36.32	34.55	37.05	39.77	39.39	40.77	40.82	40.33	35.96	35.01	35.56	40.46	45.13	47.73	47.72
Gleason grade *															
≤6	189.87	176.24	190.26	194.00	166.20	126.76	170.01	167.11	128.21	113.39	96.26	94.45	93.51	93.62	89.24
7	120.81	117.62	138.04	151.24	146.96	119.66	121.02	123.88	103.54	100.79	94.82	101.98	111.77	117.81	119.60
8-10	40.25	40.30	44.67	45.15	42.10	34.57	44.74	44.73	41.38	43.34	43.34	46.79	50.10	55.22	53.42

Delay-Adjusted Prostate Cancer Incidence (per 100 000 Men) and in men >/=75 old

	2004	2005	2006	2007	2008	2009	2010	2011	2012	2013	2014	2015	2016	2017	2018
No. of patients	10,904	10,374	11,048	11,009	9,510	8,855	8,650	8,388	6,939	6,820	6,631	7,128	7,396	7,836	8,123
PSA, ng/mL, median (IQR)	9.5	9.3	9.1	8.8	8.6	8.5	9.0	8.7	9.7	10.1	10.8	11.2	11.0	11.0	10.9
	6.1-19.3	5.9-18.3	5.9-17.7	5.6-16.7	5.5-16.95	5.5-17	5.7-18.45	5.7-17.6	44371.0	6.2-26.4	6.4-29.4	6.7-30.1	6.6-30	6.6-27.4	6.6-29.3
Incidence rate Invasive PCa	804.22	757.11	799.6	792.19	682.41	634.48	618.36	598.02	490.46	476.48	456.96	485.62	498.67	522.33	535.25
Incidence rate Localized PCa	638.21	596.08	635.26	623.37	520.56	474.92	454.6	453.19	343.37	324.35	299.98	313.59	312.77	326.23	343.24
Incidence rate Localized + regional	670.48	624.4	666.32	654.55	551.24	505.45	489.52	484.86	372.99	351.29	331.28	350.07	354.83	370.64	389.49
Localized/metastatic PCa	10.69	10.00	10.08	10.35	9.37	8.66	7.92	8.20	5.26	4.74	4.21	3.79	3.85	3.69	3.73
Metastasis *															
Summary Stage (distant)	60.27	60.42	63.9	61.16	56.64	55.6	57.88	55.66	65.58	68.93	71.8	83.31	82.67	91.52	92.66
AJCC M1	59.71	59.58	63.01	60.22	55.57	54.85	57.39	55.3	65.24	68.45	71.2	82.78	81.25	88.41	92
AJCC N1	8.94	8.9	10.18	11.97	13.24	12.45	15.14	15.87	17.71	19.68	23.44	27.12	31.43	33.16	37.01
T Stage *															
T1-T2	667.89	625.37	667.64	657.76	550.49	501.88	483.93	483.78	375.82	358.93	332.86	352.17	335.09	352.48	384.37
T3-T4	40.89	35.85	37.56	35.25	34.92	34.92	39.5	35.28	35.22	33.16	40.01	46.35	51.73	54.74	54.96
Gleason grade *															
≤6	285.52	262.05	266.52	254.49	203.06	137.50	153.01	156.88	104.74	102.75	84.33	80.28	82.56	81.92	78.47
7	223.98	218.88	237.06	249.91	214.14	145.68	193.17	192.93	140.74	132.88	121.07	137.62	140.79	151.92	155.01
8-10	166.09	155.60	176.27	168.22	145.52	108.19	159.21	152.30	137.30	130.44	141.23	152.28	166.25	174.89	178.51

*** Rates are per 100,000 and age-adjusted to the 2000 US Std Population standard**

© 2022 Desai MM et al. *JAMA Network Open.*

eTable 2. *Yearly Variations in Prostate Cancer Presentation at Diagnosis - SEER Database 2004-2018- Non-Hispanic Black. * Rates are per 100,000 and age-adjusted to the 2000 US Std Population standard*

Delay-Adjusted Prostate Cancer Incidence (per 100 000 Men) in men < 75 old															
	**2004**	**2005**	**2006**	**2007**	**2008**	**2009**	**2010**	**2011**	**2012**	**2013**	**2014**	**2015**	**2016**	**2017**	**2018**
**No. of patients**	6,052	6,018	6,594	7,178	7,156	7,590	7,463	7,673	6,967	6,835	6,449	6,969	7,421	7,820	7,752
**PSA, ng/mL, median (IQR)**	7.1	7.0	6.9	6.8	6.6	6.7	6.8	6.6	6.9	7.0	7.4	7.6	7.6	7.7	7.8
	5-12.5	5-12.8	4.9-12.4	4.8-11.7	4.8-11.6	4.8-11.6	4.9-12	4.8-11.2	4.9-12.2	44329.0	5.2-14	5.3-14	5.3-14.1	5.3-14.3	5.4-15.1
**Incidence rate Invasive PCa**	619.2	599.47	632	658.39	625.98	636.1	603.02	598.26	528.05	501.38	455.75	476.63	497.21	514.75	512.58
**Incidence rate Localized PCa**	505.08	489.5	528.26	545.95	522.73	522.92	490.37	488.92	422.81	401.49	354.32	370.84	368.99	386.3	389.05
**Incidence rate Localized + regional**	577.32	551.01	587.28	611.91	581.69	587.86	555.82	548.69	479.4	453.79	405.99	428.03	429.9	448	447.36
**Localized/metastatic PCa**	19.09	17.23	17.91	20.00	19.02	19.70	17.22	16.50	14.37	12.76	11.16	12.03	10.45	10.94	9.90
**Metastasis ***															
**Summary Stage (distant)**	27.02	29.22	29.84	27.84	27.68	26.91	28.62	29.77	29.43	31.68	31.89	31.08	36.63	36.22	39.68
**AJCC M1**	26.46	28.41	29.5	27.3	27.48	26.54	28.47	29.64	29.43	31.47	31.75	30.83	35.32	35.32	39.29
**AJCC N1**	9.78	12.42	13.39	13.56	11.83	15.51	17.41	14.03	16.01	16.99	20.41	22.53	26.46	28.37	28.16
**T Stage ***															
**T1-T2**	544.82	522.74	561.55	581.57	555.46	557.25	520.87	519.76	451.35	430.63	381.97	397.3	388.49	383.98	409.38
**T3-T4**	53.14	50.82	46.26	51.1	47.27	51.3	56.42	51.1	49.59	46.19	47.38	52.4	61.31	64.66	61.45
**Gleason grade ***															
**≤6**	284.36	262.43	269.11	269.84	247.35	196.52	239.74	240.25	193.95	174.92	147.15	150.15	148.72	147.27	140.27
**7**	209.19	213.98	232.07	256.12	242.97	212.65	234.55	228.29	210.57	203.22	184.56	198.16	211.95	219.69	221.24
**8-10**	83.35	85.53	92.82	90.57	85.23	75.43	94.19	92.51	88.55	86.20	87.76	94.15	102.98	107.31	103.14

Delay-Adjusted Prostate Cancer Incidence (per 100 000 Men) and in men >/=75 old															
	**2004**	**2005**	**2006**	**2007**	**2008**	**2009**	**2010**	**2011**	**2012**	**2013**	**2014**	**2015**	**2016**	**2017**	**2018**
**No. of patients**	1,454	1,293	1,224	1,309	1,229	1,205	1,164	1,143	1,018	981	907	936	1,029	1,105	1,083
**PSA, ng/mL, median (IQR)**	13.0	12.9	11.9	11.0	11.5	10.7	11.7	11.0	12.3	11.8	14.5	14.3	14.0	13.6	14.0
	7.4-34.7	7.2-37.4	6.75-28.4	6.5-32.8	6.6-33.7	6.5-31.4	6.6-35.2	6.2-30.4	6.8-36	7-38.8	7.7-57.5	7.7-61	7.8-52	7.2-46.8	7.7-49.5
**Incidence rate Invasive PCa**	1,315.02	1,149.24	1,067.52	1,122.70	1,034.01	986.41	925.58	878.81	757.83	710.86	637.91	634.53	673.99	705.14	676.78
**Incidence rate Localized PCa**	958.08	840.2	801.52	842.16	746.99	678.47	635.45	636.34	517.62	467.23	384.73	388.44	390.92	411.24	437.49
**Incidence rate Localized + regional**	991.82	872.09	832.78	873.1	782.26	708.18	671.43	661.29	539.23	493.6	409.46	415.16	425.71	445.73	472.67
**Localized/metastatic PCa**	6.74	6.27	7.37	8.03	7.17	5.78	5.76	6.29	5.34	4.53	3.57	3.26	3.12	2.83	3.53
**Metastasis ***															
**Summary Stage (distant)**	143.94	133.92	108.72	106.81	105.96	117.29	110.41	101.9	96.92	103.87	109.17	119.76	123.82	150	125.51
**AJCC M1**	142.24	133.92	108.72	104.94	104.15	117.29	110.41	101.18	96.92	103.19	107.63	118.99	125.24	145.26	124.11
**AJCC N1**	15.74	19.62	18.8	20.64	22.72	25.75	19.53	23.91	21.05	20.05	33.8	32.01	33.33	50.06	37.93
**T Stage ***															
**T1-T2**	1,028.01	912.84	847.05	893.79	799.35	735.95	691.25	693.82	574.18	512.53	428.78	430.58	432.56	436.65	491.33
**T3-T4**	55.32	54.86	50.94	49.5	55.47	41.66	51.53	33.11	27.17	45.64	40.88	41.22	49.38	55.46	45.69
**Gleason grade ***															
**≤6**	364.32	338.23	296.04	306.61	269.69	194.44	227.79	197.17	167.96	141.13	94.89	101.24	102.58	105.42	110.56
**7**	402.02	312.30	314.01	360.81	317.64	242.12	284.41	294.32	216.74	193.61	178.05	171.40	198.55	203.04	198.33
**8-10**	254.07	251.25	237.50	234.42	224.40	166.20	218.30	212.00	184.44	188.14	161.85	177.79	209.19	198.90	208.40

*** Rates are per 100,000 and age-adjusted to the 2000 US Std Population standard**

© 2022 Desai MM et al. *JAMA Network Open.*

*eTable 3. Yearly Variations in Prostate Cancer Presentation at Diagnosis - SEER Database 2004-2018- Hispanic. * Rates are per 100,000 and age-adjusted to the 2000 US Std Population standard*

Delay-Adjusted Prostate Cancer Incidence (per 100 000 Men) in men < 75 old

	2004	2005	2006	2007	2008	2009	2010	2011	2012	2013	2014	2015	2016	2017	2018
No. of patients	3,474	3,406	3,633	3,932	3,999	4,323	4,194	4,391	3,991	3,884	3,857	4,125	4,261	4,583	4,439
PSA, ng/mL, median (IQR)	7.1	6.7	7.0	6.7	6.6	6.5	6.6	6.5	6.7	7.0	7.2	7.3	7.3	7.4	7.5
	5.1-11.7	4.9-11	5-11.6	4.9-11	4.8-10.8	4.8-10.5	4.9-10.5	4.8-10.2	4.9-10.6	5.1-11.8	5.1-12.7	5.2-13	5.2-13	5.3-13	5.4-13.1
Incidence rate Invasive PCa	325.19	298.8	304.52	311.01	298.33	304.19	279.01	278.57	239.04	221.29	206.97	209.9	207.54	214.45	202.3
Incidence rate Localized PCa	256.33	234.39	244.26	240.54	229.26	231.88	211.4	213.22	182.16	162.27	148.63	148.04	141.33	141.87	134.52
Incidence rate Localized + regional	301.63	275.63	282.99	281.51	269.37	272.6	249.42	250.06	212.44	192.28	179.35	181.96	171.87	173.21	166.06
Localized/metastatic PCa	19.96	16.21	19.87	18.87	20.15	17.05	18.94	17.21	13.63	11.50	10.82	10.74	9.52	10.03	8.72
Metastasis *															
Summary Stage (distant)	13.64	14.62	12.29	12.92	11.52	13.68	11.27	12.59	13.41	14.11	13.97	14.02	15.3	14.5	15.71
AJCC M1	12.84	14.46	12.29	12.75	11.38	13.6	11.16	12.39	13.36	14.11	13.74	13.78	14.85	14.15	15.42
AJCC N1	6.8	6.74	6.75	7.17	8.1	7.17	7.3	8.05	8.46	8.7	11.08	10.94	12.34	11.83	12.38
T Stage *															
T1-T2	279.78	256.23	263.41	261.09	246.69	247.78	226.93	227.26	195.32	174.68	160.91	159.7	148.2	146.1	141.44
T3-T4	32.47	29.17	28.42	30.15	30.17	33.21	31.18	31.41	26.14	27.71	28.38	31.6	30.67	32.23	31.83
Gleason grade *															
≤6	163.11	142.46	135.23	139.26	124.44	102.04	131.90	130.31	107.31	91.65	81.30	74.41	69.09	70.87	62.01
7	97.17	95.63	104.69	106.54	108.62	85.82	86.57	89.51	77.83	73.78	70.52	74.62	78.33	76.25	72.01
8-10	42.12	37.47	41.48	42.62	41.90	33.74	41.50	39.38	36.07	37.71	37.89	41.77	41.97	43.32	40.50

Delay-Adjusted Prostate Cancer Incidence (per 100 000 Men) and in men >/=75 old

	2004	2005	2006	2007	2008	2009	2010	2011	2012	2013	2014	2015	2016	2017	2018
No. of patients	3,474	3,406	3,633	3,932	3,999	4,323	4,194	4,391	3,991	3,884	3,857	4,125	4,261	4,583	4,439
PSA, ng/mL, median (IQR)	11.0	10.5	10.0	9.6	9.6	9.9	10.4	10.6	12.0	11.8	11.8	14.1	13.0	15.2	13.6
	6.8-26	6.6-22.1	6.1-20.4	6.3-20	6.2-20.45	6.3-22.1	6.6-26.5	6.4-24.4	7-31.9	6.75-41.25	6.9-34	7.3-51.5	7.1-37.5	7.95-57.2	7.4-46.9
Incidence rate Invasive PCa	897.96	855.3	795.11	784.74	764.52	671.52	660.11	575.63	512.3	513.58	442.82	446.98	451.28	472.6	458.9
Incidence rate Localized PCa	675.86	622.73	601.73	567.1	542.83	447.62	435.93	394.92	338.16	319.82	264.66	255.8	253.37	248.66	249.54
Incidence rate Localized + regional	706.94	657.85	642.79	600.94	567.98	475.96	466.66	417.8	367.7	350.65	290.11	287.51	280.48	278.45	285.07
Localized/metastatic PCa	7.51	8.27	9.81	10.06	8.04	7.45	6.04	5.83	5.25	4.47	4.37	3.49	3.56	2.96	2.80
Metastasis *															
Summary Stage (distant)	91.53	76.03	61.98	56.97	68.16	60.1	73.43	67.75	65.51	71.56	60.52	73.33	72.68	86.99	90.49
AJCC M1	90.05	75.3	61.34	56.35	67.53	60.1	72.18	67.75	64.41	71.56	60.52	73.33	71.26	83.93	89.2
AJCC N1	^	14.17	12.68	11.79	10.65	16.22	15.4	15.18	22.16	21.62	18.58	25.69	26.62	33.67	39
T Stage *															
T1-T2	711.75	663.62	633.95	592.71	573.99	477.71	475.65	423.25	363.92	352.47	293.36	291.44	265.62	276.69	278.35
T3-T4	44.3	41.63	45.77	39.51	34.52	37.19	39.66	25.42	38.19	37.09	30.42	36.4	37.99	32.93	49.67
Gleason grade *															
≤6	302.55	281.25	265.90	273.18	228.09	141.28	196.59	168.30	120.59	113.92	83.44	63.61	78.62	70.91	68.40
7	263.67	227.07	231.05	215.57	238.22	133.05	194.83	172.03	141.15	128.42	115.54	128.00	127.50	115.68	102.66
8-10	161.11	174.95	158.98	167.79	160.73	112.86	155.25	133.59	134.45	155.55	130.19	134.72	132.26	157.16	146.53

*** Rates are per 100,000 and age-adjusted to the 2000 US Std Population standard**

^ Statistic not displayed due to fewer than 16 cases.

© 2022 Desai MM et al. *JAMA Network Open.*

***eTable 4**. Age-Adjusted metastatic Prostate Cancer rates and Joinpoint analysis (SEER 18, 2004-2018)*

		Segment start	Segment End	APC	APC 95% LCL	APC 95% UCL	Test Statistic	P-Value
**Invasive Prostate Cancer, SEER 18 Registries, 2004-2018 Derived AJCC Summary stage (distant)**								
**All Races**	**45-74**	2004	2010	-0.4	-1.7	1.1	-0.6	0.585
		2010	2018	5.3	4.5	6	15.9	<0.001
	**≥75 yrs.**	2004	2011	-1.5	-3	0	-2.3	0.046
		2011	2018	6.5	5.1	7.8	11.1	<0.001
**Non-Hispanic White**	**45-74**	2004	2012	1.4	0	2.8	2.3	0.047
		2012	2018	7.5	5.7	9.3	9.6	<0.001
	**≥75 yrs.**	2004	2010	-1.7	-4.2	0.8	-1.5	0.164
		2010	2018	6.9	5.4	8.4	10.5	<0.001
**Non-Hispanic Black**	**45-74**	2004	2012	0.5	-1.3	2.3	0.6	0.562
		2012	2018	5	2.7	7.3	5	0.001
	**≥75 yrs.**	2004	2012	-3.7	-6.7	-0.5	-2.6	0.026
		2012	2018	6.2	1.6	11.1	3	0.013
**Hispanic**	**45-74**	2004	2018	1.3	0.3	2.3	2.9	0.012
	**≥75 yrs.**	2004	2006	-20.6	-44	12.4	-1.5	0.17
		2006	2018	3	1.2	4.8	3.7	0.004
**Invasive Prostate Cancer, SEER 18 Registries, 2004-2018 Derived AJCC M stage (M1)**								
**All Races**	**45-74 yrs.**	2004	2010	-0.1	-1.5	1.2	-0.2	0.817
		2010	2018	5.1	4.4	5.9	16	<0.001
	**≥75 yrs.**	2004	2010	-2.3	-4.2	-0.4	-2.7	0.022
		2010	2018	5.6	4.5	6.8	11.6	<0.001
**Hispanic**	**45-74 yrs.**	2004	2018	1.3	0.4	2.3	3	0.01
	**≥75 yrs.**	2004	2006	-20.1	-42.7	11.3	-1.5	0.162
		2006	2018	2.9	1.1	4.6	3.7	0.004
**non-Hispanic Black**	**45-74 yrs.**	2004	2015	1.5	0.5	2.5	3.5	0.006
		2015	2018	7.6	1.8	13.6	3	0.014
	**≥75 yrs.**	2004	2012	-3.6	-6.6	-0.6	-2.6	0.025
		2012	2018	6	1.4	10.7	3	0.014
**non-Hispanic White**	**45-74 yrs.**	2004	2009	-0.8	-3.7	2.1	-0.6	0.546
		2009	2018	5.9	4.9	6.9	13.2	<0.001
	**≥75 yrs.**	2004	2010	-1.6	-4.1	1	-1.4	0.191
		2010	2018	6.8	5.3	8.3	10.4	<0.001
**Invasive Prostate Cancer, SEER 18 Registries, 2004-2018 Derived AJCC N stage (N1)**								
**All Races**	**45-74 yrs.**	2004	2012	3.4	1.6	5.3	4.1	0.002
		2012	2018	10.4	8.2	12.6	11	<0.001
	**≥75 yrs.**	2004	2007	12.6	6.6	18.9	27.6	0.023
		2007	2010	4.9	-4.2	14.9	6.7	0.094
		2010	2013	10	1.6	19	15.3	0.041
		2013	2016	14.8	7.7	22.4	27.4	0.023
		2016	2018	8.8	3.2	14.7	20.4	0.031
**Hispanic**	**45-74 yrs.**	2004	2018	5.4	4.3	6.5	10.9	<0.001
	**≥75 yrs.**	2004	2018	10	8	12.2	10.8	<0.001
**non-Hispanic Black**	**45-74 yrs.**	2004	2018	7.6	6.1	9	11.7	<0.001
	**≥75 yrs.**	2004	2018	7.1	4.6	9.7	6.3	<0.001
**non-Hispanic White**	**45-74 yrs.**	2004	2012	3.2	1.4	5.1	4	0.003
		2012	2018	11.2	9	13.5	11.7	<0.001
	**≥75 yrs.**	2004	2018	11.5	10.6	12.4	29.7	<0.001
**Invasive Prostate Cancer, SEER 18 Registries, 2004-2018 Derived AJCC T stage (T3-T4)**								
**All Races**	**45-74 yrs.**	2004	2010	2.3	-0.4	5.1	2	0.085
		2010	2013	-5.7	-18.8	9.4	-0.9	0.379
		2013	2018	6.5	3.3	9.8	4.9	0.002
	**≥75 yrs.**	2004	2012	-2.2	-4.1	-0.4	-2.6	0.025
		2012	2018	7.9	5.1	10.8	6.5	<0.001
**Hispanic**	**45-74 yrs.**	2004	2018	0.2	-0.7	1.1	0.5	0.601
	**≥75 yrs.**	2004	2018	-0.2	-2.3	1.9	-0.3	0.805
**non-Hispanic Black**	**45-74 yrs.**	2004	2014	-0.3	-2.1	1.5	-0.4	0.71
		2014	2018	7.4	1.1	14	2.6	0.025
	**≥75 yrs.**	2004	2018	-0.9	-3.1	1.3	-0.9	0.377
**non-Hispanic White**	**45-74 yrs.**	2004	2010	2.8	0.2	5.4	2.6	0.037
		2010	2013	-5.9	-18.3	8.3	-1	0.338
		2013	2018	7.5	4.4	10.7	5.9	0.001
	**≥75 yrs.**	2004	2013	-1.1	-2.9	0.6	-1.5	0.168
		2013	2016	15.1	-3.3	37.2	1.9	0.098
		2016	2018	2.5	-12	19.4	0.4	0.71

© 2022 Desai MM et al. *JAMA Network Open.*

***eTable 5**. Age-Adjusted metastatic Prostate Cancer rates and Joinpoint analysis (SEER 18, 2004-2018). Joinpoint analysis modeled for periods 2004-2010 and 2010-2018.*

Segment Start-End	Race/ethnicity	Age	APC	APC 95% LCL	APC 95% UCL	Test Statistic	P-Value	P-Value	Parallelism
**Invasive Prostate Cancer, SEER 18 Registries, 2004-2018 Derived AJCC Summary stage (distant)**									
2004-2010	All Races	45-74	-0.1	-1.2	1.0	-0.3	0.811		
		75+	-2.0	-2.9	-0.9	-5.0	0.004	0.016	Rejected
	Hispanic	45-74	-2.6	-6.6	1.5	-1.7	0.159		
		75+	-3.4	-10.2	3.8	-1.2	0.267	0.964	Failed to reject
	Non-Hispanic Black	45-74	-0.3	-2.3	1.8	-0.4	0.726		
		75+	-3.9	-8.1	0.5	-2.3	0.069	0.236	Failed to reject
	Non-Hispanic White	45-74	0.3	-1.2	1.8	0.5	0.642		
		75+	-1.5	-3.4	0.5	-1.9	0.114	0.023	Rejected
2010-2018	All Races	45-74	5.2	4.4	6.0	15.5	<0.001		
		75+	5.8	4.4	7.2	10.4	<0.001	0.436	Failed to reject
	Hispanic	45-74	3.2	1.9	4.6	5.7	0.001		
		75+	3.5	0.5	6.7	2.7	0.029	0.867	Failed to reject
	Non-Hispanic Black	45-74	4.1	2.7	5.5	7.0	<0.001		
		75+	4.1	1.2	7.1	3.4	0.012	0.978	Failed to reject
	Non-Hispanic White	45-74	6.2	5.1	7.5	12.7	<0.001		
		75+	6.9	5.5	8.2	12.2	<0.001	0.633	Failed to reject
**Invasive Prostate Cancer, SEER 18 Registries, 2004-2018 Derived AJCC M stage (M1)**									
2004-2010	All Races	45-74	0.0	-1.2	1.2	0.0	0.976		
		75+	-1.9	-3.0	-0.9	-4.6	0.006	0.007	Rejected
	Hispanic	45-74	-2.2	-6.3	2.2	-1.3	0.255		
		75+	-3.4	-10.0	3.6	-1.3	0.261	0.819	Failed to reject
	Non-Hispanic Black	45-74	0.0	-2.1	2.1	0.0	0.992		
		75+	-3.8	-8.2	0.7	-2.2	0.082	0.212	Failed to reject
	Non-Hispanic White	45-74	0.4	-1.3	2.0	0.6	0.599		
		75+	-1.5	-3.4	0.5	-1.9	0.118	0.031	Rejected
2010-2018	All Races	45-74	5.0	4.3	5.8	16.8	<0.001		
		75+	5.6	4.3	6.9	10.8	<0.001	0.453	Failed to reject
	Hispanic	45-74	3.0	1.5	4.4	4.9	0.002		
		75+	3.3	0.4	6.3	2.7	0.03	0.843	Failed to reject
	Non-Hispanic Black	45-74	3.8	2.5	5.2	6.9	<0.001		
		75+	3.9	1.2	6.7	3.4	0.011	0.938	Failed to reject
	Non-Hispanic White	45-74	6.1	4.9	7.2	12.4	<0.001		
		75+	6.7	5.3	8.0	12.0	<0.001	0.653	Failed to reject
**Invasive Prostate Cancer, SEER 18 Registries, 2004-2018 Derived AJCC N stage (N1)**									
2004-2010	All Races	45-74	3.6	2.5	4.8	8.4	<0.001		
		75+	8.3	5.5	11.2	7.8	0.001	0.016	Rejected
	Hispanic	45-74	1.8	-1.2	4.9	1.6	0.18		
		75+	6.3	-2.5	15.9	1.8	0.129	0.239	Failed to reject
	Non-Hispanic Black	45-74	7.7	2.5	13.2	3.8	0.012		
		75+	5.0	-1.4	11.8	2.0	0.103	0.640	Failed to reject
	Non-Hispanic White	45-74	3.1	1.6	4.7	5.2	0.004		
		75+	9.4	6.0	12.9	7.2	0.001	<0.001	Rejected
2010-2018	All Races	45-74	9.2	7.3	11.2	11.6	<0.001		
		75+	12.0	11.1	13.0	30.6	<0.001	0.098	Failed to reject
	Hispanic	45-74	7.0	4.7	9.3	7.4	<0.001		
		75+	12.2	8.2	16.2	7.7	<0.001	0.108	Failed to reject
	Non-Hispanic Black	45-74	9.3	6.2	12.5	7.3	<0.001		
		75+	11.3	5.2	17.7	4.5	0.003	0.602	Failed to reject
	Non-Hispanic White	45-74	9.8	7.8	11.9	12.0	<0.001		
		75+	12.8	11.5	14.2	23.2	<0.001	0.114	Failed to reject
**Invasive Prostate Cancer, SEER 18 Registries, 2004-2018 Derived AJCC T stage (T3-T4)**									
2004-2010	All Races	45-74	2.2	0.8	3.7	4.0	0.011		
		75+	-1.2	-3.5	1.1	-1.3	0.238	0.007	Rejected
	Hispanic	45-74	0.8	-2.0	3.7	0.7	0.49		
		75+	-2.8	-6.5	1.0	-1.9	0.114	0.041	Rejected
	Non-Hispanic Black	45-74	1.0	-2.3	4.5	0.8	0.479		
		75+	-2.1	-6.3	2.3	-1.3	0.264	0.306	Failed to reject
	Non-Hispanic White	45-74	2.6	1.1	4.2	4.5	0.006		
		75+	-0.8	-4.0	2.5	-0.6	0.559	0.038	Rejected
2010-2018	All Races	45-74	2.5	-0.2	5.2	2.1	0.069		
		75+	5.7	2.7	8.7	4.6	0.002	0.116	Failed to reject
	Hispanic	45-74	1.1	-1.0	3.2	1.3	0.25		
		75+	3.0	-2.3	8.6	1.3	0.222	0.476	Failed to reject
	Non-Hispanic Black	45-74	2.9	-0.2	6.1	2.2	0.063		
		75+	3.0	-2.9	9.2	1.2	0.278	0.972	Failed to reject
	Non-Hispanic White	45-74	3.1	0.2	6.0	2.5	0.041		
		75+	6.5	3.4	9.8	4.9	0.002	0.086	Failed to reject

© 2022 Desai MM et al. *JAMA Network Open.*

Supplementary figures

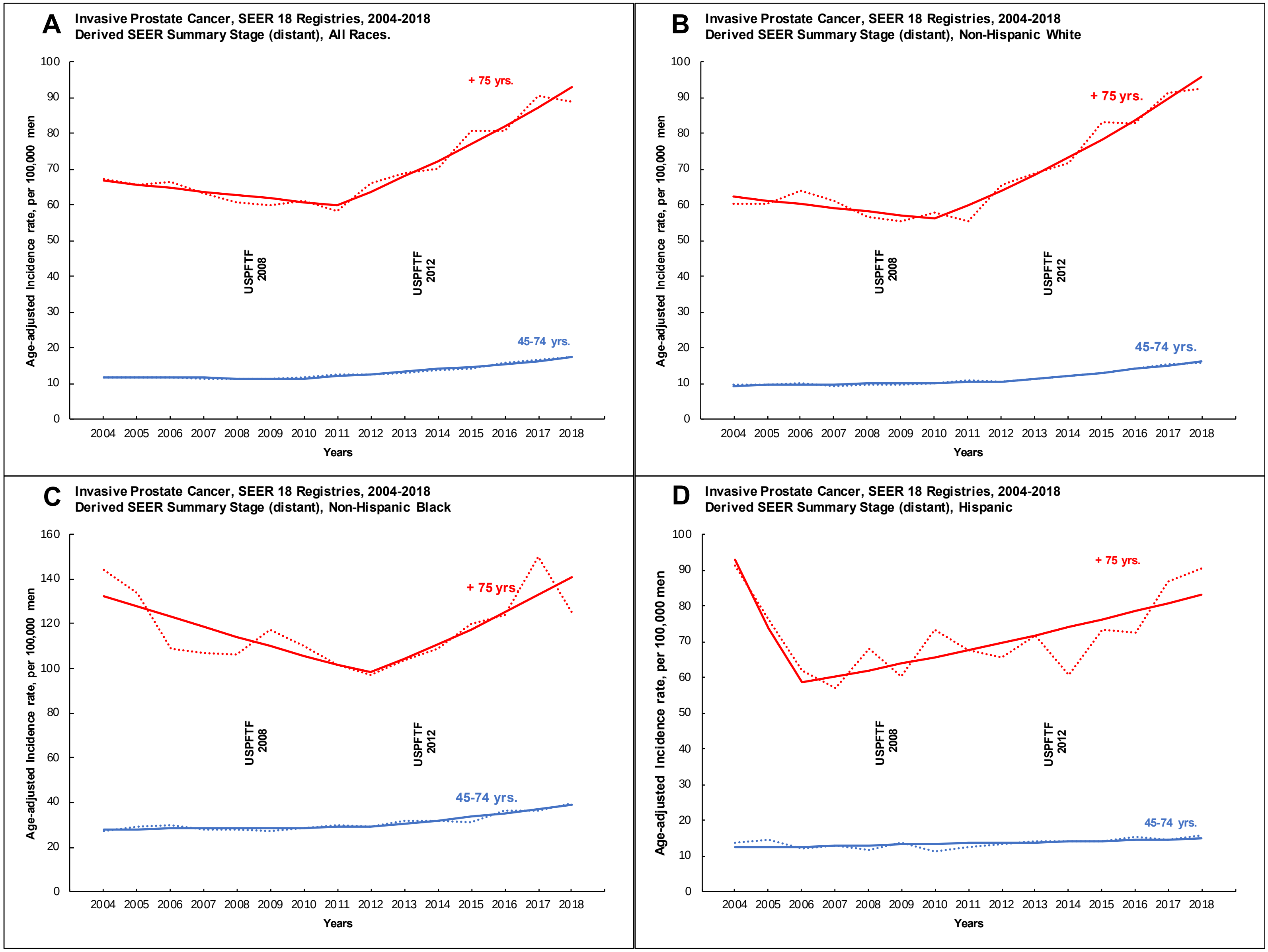

e**Figure 1.** Trends in malignant prostate cancer, SEER 18 Registries 2004-2018. Derived SEER summary stage (distant) by race a) All races, b) Non-Hispanic White c) Non-Hispanic Black d) Hispanic. *Continue line: modeled count; Dotted line: Delay age-adjusted rate*

© 2022 Desai MM et al. *JAMA Network Open.*

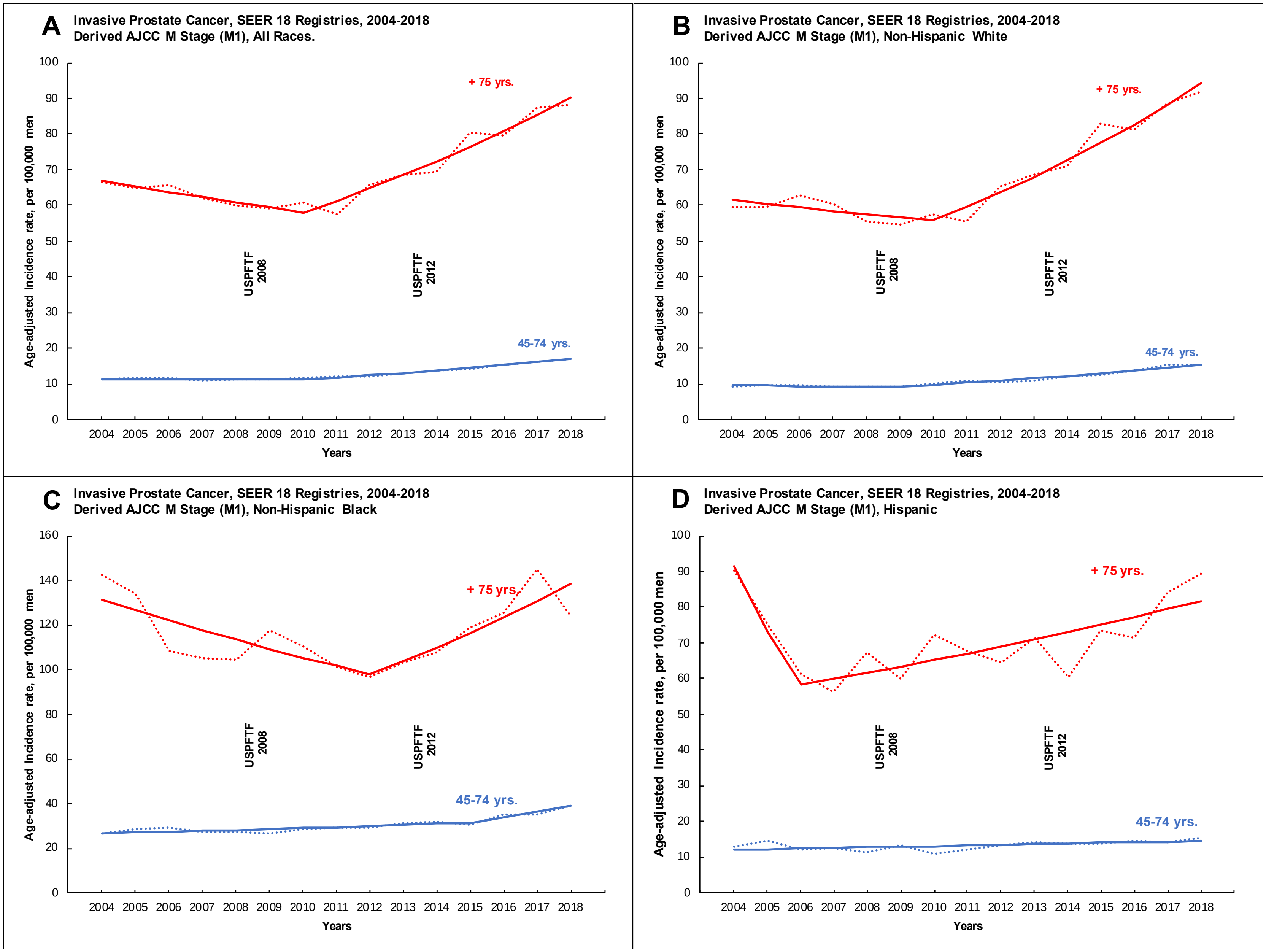

e**Figure 2.** Trends in malignant prostate cancer, SEER 18 Registries 2004-2018. Derived AJCC M stage (M1) by race a) All races, b) Non-Hispanic White c) Non-Hispanic Black d) Hispanic. *Continue line: modeled count; Dotted line: Delay age-adjusted rate*

© 2022 Desai MM et al. *JAMA Network Open.*

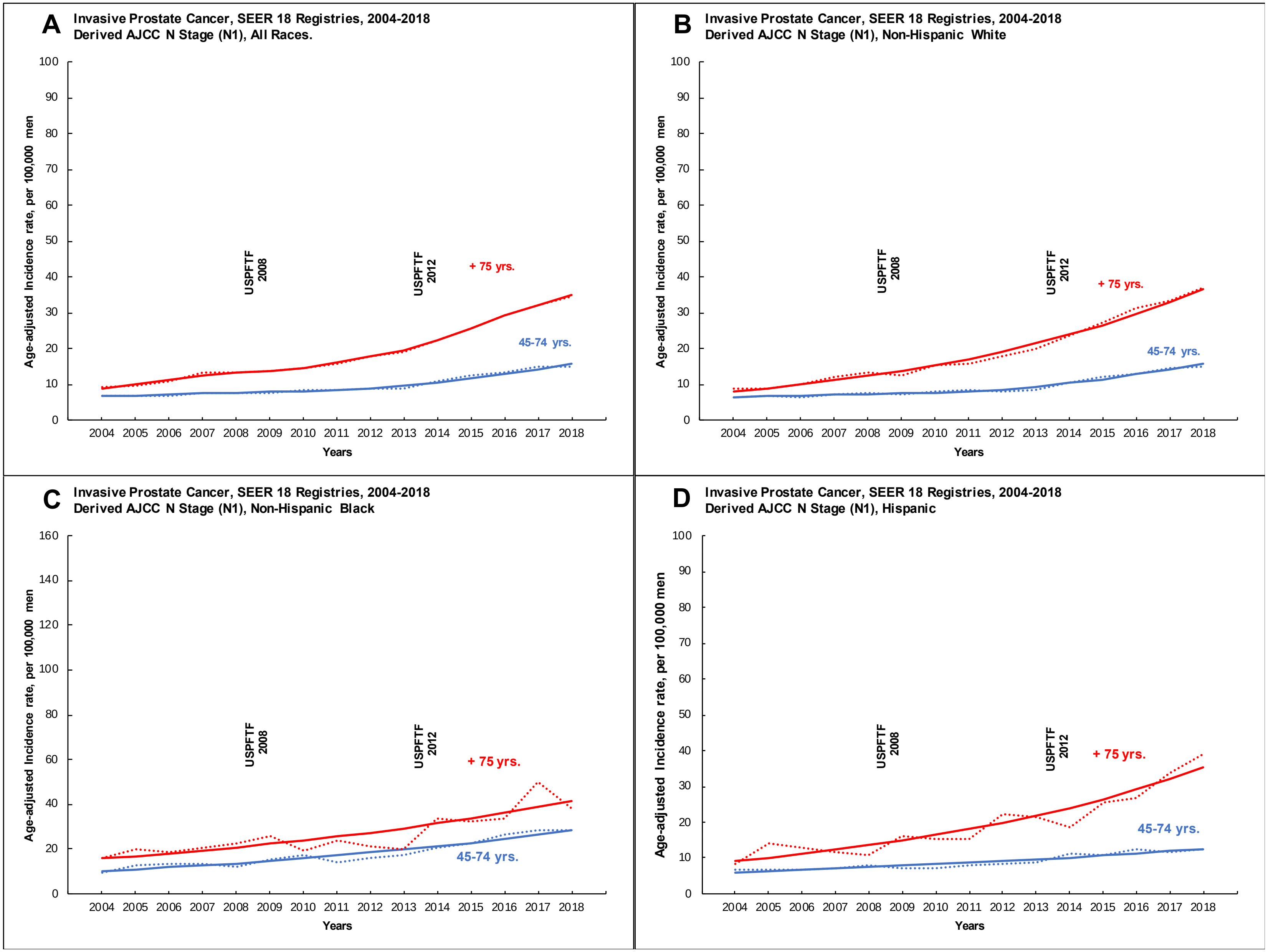

eFigure 3. Trends in malignant prostate cancer, SEER 18 Registries 2004-2018. Derived AJCC N stage (N1) by race a) All races, b) Non-Hispanic White c) Non-Hispanic Black d) Hispanic. *Continue line: modeled count; Dotted line: Delay age-adjusted rate*

© 2022 Desai MM et al. *JAMA Network Open.*

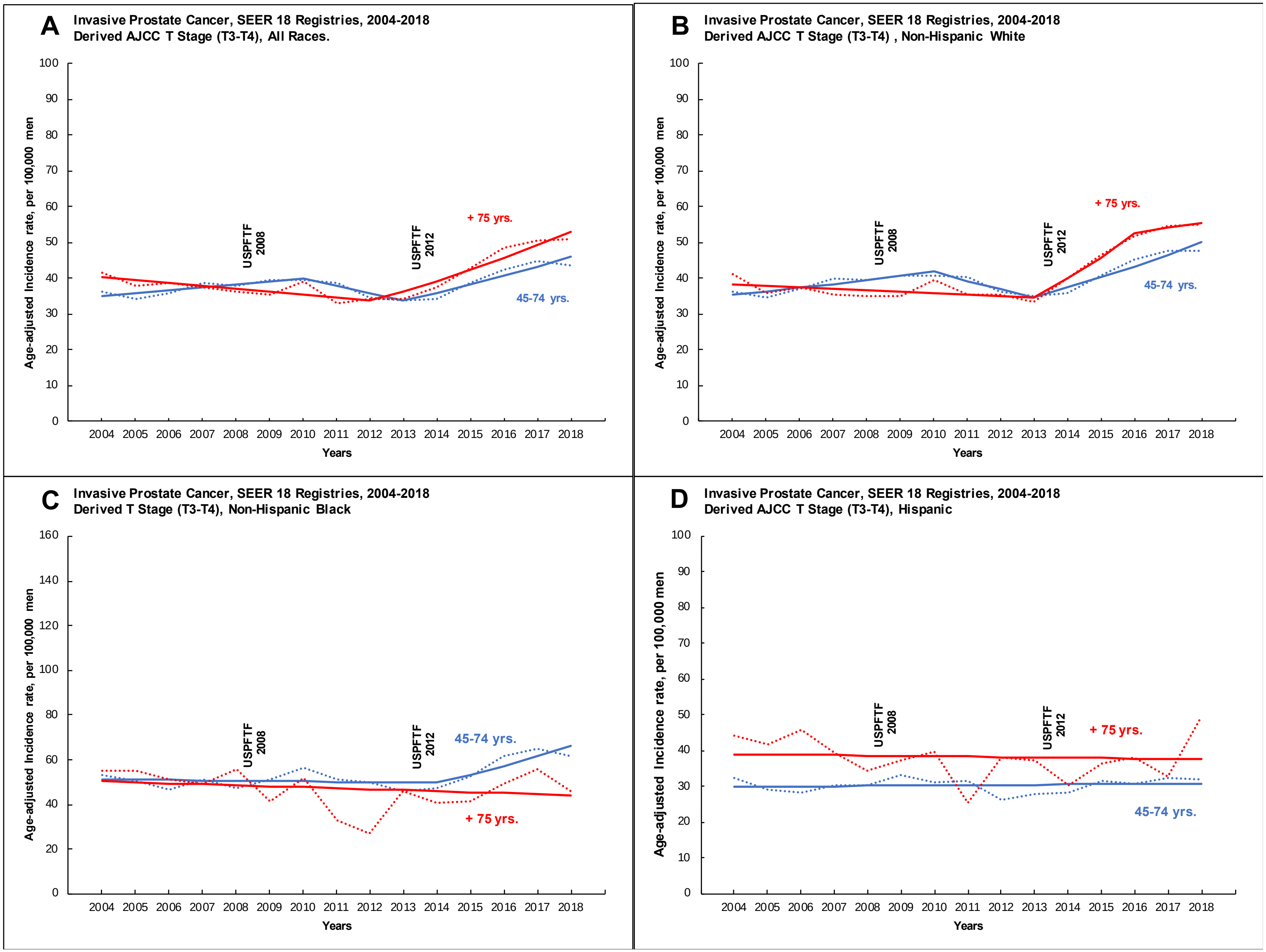

e**Figure 4.** Trends in malignant prostate cancer, SEER 18 Registries 2004-2018. Derived AJCC T stage (T3-T4) by race a) All races, b) Non-Hispanic White c) Non-Hispanic Black d) Hispanic. *Continue line: modeled count; Dotted line: Delay age-adjusted rate*

© 2022 Desai MM et al. *JAMA Network Open.*
